# Carbohydrate restricted recovery from long term endurance exercise does not affect gene responses involved in mitochondrial biogenesis in highly trained athletes

**DOI:** 10.14814/phy2.12184

**Published:** 2015-02-12

**Authors:** Line Jensen, Kasper D Gejl, Niels Ørtenblad, Jakob L Nielsen, Rune D Bech, Tobias Nygaard, Kent Sahlin, Ulrik Frandsen

**Affiliations:** 1 Institute of Sports Science and Clinical Biomechanics, SDU Muscle Research Cluster, University of Southern DenmarkOdense, Denmark; 2 Institute of Clinical Research, Clinical Pathology, SDU Muscle Research Cluster, University of Southern DenmarkOdense, Denmark; 3 Department of Health Sciences, Swedish Winter Sports Research Centre, Mid Sweden UniversityÖstersund, Sweden; 4 Department of Orthopedic Surgery, Odense University HospitalOdense, Denmark; 5 Department of Orthopedic Surgery, RigshospitaletCopenhagen, Denmark; 6 The Åstrand Laboratory of Work Physiology, GIH, The Swedish School of Sport and Health SciencesStockholm, Sweden

**Keywords:** baseline mRNA expression, glycogen depletion, PGC-1*α*, type 1 fibers

## Abstract

The aim was to determine if the metabolic adaptations, particularly PGC-1*α* and downstream metabolic genes were affected by restricting CHO following an endurance exercise bout in trained endurance athletes. A second aim was to compare baseline expression level of these genes to untrained. Elite endurance athletes (VO_2max_ 66 ± 2 mL·kg^−1^·min^−1^, *n* = 15) completed 4 h cycling at ∼56% VO_2max_. During the first 4 h recovery subjects were provided with either CHO or only H_2_O and thereafter both groups received CHO. Muscle biopsies were collected before, after, and 4 and 24 h after exercise. Also, resting biopsies were collected from untrained subjects (*n* = 8). Exercise decreased glycogen by 67.7 ± 4.0% (from 699 ± 26.1 to 239 ± 29.5 mmol·kg^−1^·dw^−1^) with no difference between groups. Whereas 4 h of recovery with CHO partly replenished glycogen, the H_2_O group remained at post exercise level; nevertheless, the gene expression was not different between groups. Glycogen and most gene expression levels returned to baseline by 24 h in both CHO and H_2_O. Baseline mRNA expression of NRF-1, COX-IV, GLUT4 and PPAR-*α* gene targets were higher in trained compared to untrained. Additionally, the proportion of type I muscle fibers positively correlated with baseline mRNA for PGC-1*α*, TFAM, NRF-1, COX-IV, PPAR-*α*, and GLUT4 for both trained and untrained. CHO restriction during recovery from glycogen depleting exercise does not improve the mRNA response of markers of mitochondrial biogenesis. Further, baseline gene expression of key metabolic pathways is higher in trained than untrained.

## Introduction

Carbohydrate (CHO) is an important fuel for skeletal muscle both during (Bergstrom et al. 1967) and following exercise (Bergstrom and Hultman 1967), and depletion of muscle glycogen during prolonged exercise is linked to fatigue and impairment of performance in training and competition (Jacobs et al. 1981).

Within the last decade, a new concept of exercising with reduced muscle glycogen levels (“train low”) has been introduced in order to optimize the adaption to training (Pilegaard et al. 2002). Exercising with low muscle glycogen levels or restriction of CHO intake during or after exercise may be a way of increasing training induced transcriptional improvements in muscle oxidative capacity, which can potentially enhance athletic performance.

In the pioneering study by Hansen and colleges (Hansen et al. 2005) untrained (UT) men performed kicking-exercise in one leg every day, while the other leg was exercised twice every other day, the second bout performed with low glycogen availability. After 10 weeks of training time to exhaustion at 90% power output was increased in the twice-a-day leg only (294 vs. 113%, respectively). Similarly, trained men cycling twice every second day compared to once every day for 3 weeks, showed increased glycogen storage, fat oxidation and mitochondrial enzyme activity, although no improvement in performance was found (Yeo et al. 2008).

Only a few studies have investigated the effect of CHO restriction during recovery. Pilegaard et al. (2005) reported augmented activation of metabolic genes following 75 min cycling exercise combined with long term (>5 h) low CHO recovery diet when compared to controls. Mathai et al. found no differences between high or low CHO diet during recovery from 2 h cycling (Mathai et al. 2008) and Cluberton et al. supported this finding with high or low CHO diet during the first 3 h after 60 min cycling (Cluberton et al. 2005). Thus, the effect of CHO manipulation during recovery is currently uncertain.

It is a well-established fact that exercise induced changes in gene transcription and translation regulate protein adaptations through distinct signaling pathways (Pilegaard et al. 2000; Joseph et al. 2006). Consequently, repeated endurance exercise leads to adaptations in oxidative capacity, e.g. increase in size, density and efficiency of mitochondria, improved metabolic pathways and increased capillary density (Coffey and Hawley 2007). The importance of peroxisome proliferator-activated receptor gamma co-activator 1-alpha (PGC-1*α*) orchestrating key downstream events in this context is well recognized (Scarpulla 2002; Ojuka 2004; Olesen et al. 2010). Both the regulation of mitochondrial biogenesis and activation of PGC-1*α* have been linked to exercise and CHO availability through several pathways. PGC-1*α* coordinates the expression of both nuclear and mitochondrial genes by interacting with several transcription factors, including nuclear respiratory factor 1 (NRF1), which in turn activates mitochondrial transcription factor A (TFAM) (Scarpulla et al. 2012). Although the role of PGC-1*α* in regulating multiple aspects of metabolic adaptations to exercise is well established, the molecular mechanisms mediating the response are not fully understood, particularly in the highly trained (HT) (Laursen and Jenkins 2002).

Years of training leads to a highly adapted skeletal muscle phenotype (Wittwer et al. 2004) and ability to optimize fuel expenditure (Erlenbusch et al. 2005), and baseline mRNA levels potentially direct gene responses to exercise differently in HT. Most studies evaluating the effect of specific exercising regimes are conducted in UT or moderately trained subjects using short (<1 h), non-applied exercising protocols. After introducing the concepts of “train low” and CHO restriction during recovery, it is important to investigate if the CHO availability/restriction modes introduce similar effects in highly endurance trained subjects. Here we conduct long term (4 h) endurance exercise in HT subjects reflecting a real-life cycling session in regards to intensity and duration, and examine the importance of CHO availability on the expression of genes involved in metabolic adaptation. Further, baseline levels of these key metabolic cell signaling pathways have not been compared between highly endurance trained and controls.

The specific aim was to determine if the metabolic adaptations, particularly PGC-1*α* and downstream metabolic targets were affected by restricting CHO during the initial 4 h following an endurance exercise bout in HT endurance athletes. Further, baseline expression levels of these genes were compared to untrained. We hypothesized that: (1) HT athletes display higher baseline levels of key regulatory mitochondrial and metabolic genes compared to UT; (2) exhaustive cycling exercise induce an up-regulation of PGC-1*α* and downstream key regulatory metabolic genes in HT athletes; and (3) a delayed replenishment of glycogen stores during post exercise recovery further up-regulate these genes.

## Experimental Protocol

### Subjects

Fifteen healthy HT Danish triathletes were included in the study (Table1). All athletes competed at national and/or international level. Six triathletes were current or former Danish National Team members, and four were recently placed in the top three at the European or World Long Distance Triathlon Championships. One to two weeks before participating in the study the subjects visited the experimental facility to get familiarized with the exercise protocol and to determine general anthropometrics, maximal oxygen uptake (VO_2max_) and maximal heart rate (HR_max_). Eight UT control subjects were recruited for purposes of comparing baseline gene expression levels (Table1), and did not participate in the exercise protocol. All subjects were included in the study after informed written consent. The study was approved by the local ethical committee of the Region of Southern Denmark (Project-ID S-20090140) and was conducted according to the Declaration of Helsinki.

**Table 1:** Anthropometric, physiological and training characteristics of participants.

	CHO	H_2_O	HT (CHO + H_2_O)	UT
*n*	8	7	15	8
Age (years)	27.0 ± 1.0	27.1 ± 1.5	27.0 ± 0.8	22.3 ± 0.3†
Height (cm)	182 ± 2	184 ± 2	183 ± 1	181 ± 2
Body Mass (kg)	74.1 ± 1.8	77.7 ± 2.5	75.8 ± 1.5	77.9 ± 3.4
Body Fat (%)	9.6 ± 0.6	11.0 ± 0.6	10.2 ± 0.5	–
History of elite training (years)	4.6 ± 1.0	4.7 ± 1.8	4.6 ± 0.6	None
Training volume (h·week^−1^)	16.9 ± 1.4	15.3 ± 1.2	16.1 ± 0.9	0.3 ± 0.2†
VO_2max_ (L·min^−1^)	5.08 ± 0.17	4.93 ± 0.18	5.00 ± 0.12	–
VO_2max_ (mL·kg^−1^·min^−1^)	68.5 ± 1.3	63.5 ± 1.8*	66.2 ± 1.2	–
Type I fibres (%)	73.5 ± 2.7	55.4 ± 3.2*	65.0 ± 3.1	48.3 ± 5.3†
Type II fibres (%)	26.5 ± 2.9	43.6 ± 3.4*	35.0 ± 3.3	51.7 ± 5.3†

Data from CHO, H_2_O and UT groups and pooled data from all HT are presented.

* Significantly different from CHO, *P* < 0.05.

† Significantly different from HT, *P* < 0.05.

### Exercise protocol

**Figure 1:**
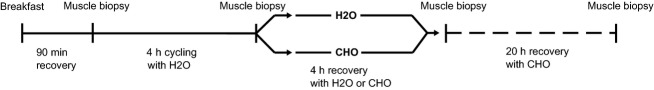
Exercise protocol designed for glycogen manipulation during recovery. Schematic illustration of the experimental design. All participants followed the same procedure, apart from receiving either water (H_2_O) or carbohydrate (CHO) during the first 4 h of recovery.

The HT subjects completed a single exercise session consisting of 4 h of cycling at an average of 73 ± 1% of HR_max_, (CHO; 74 ± 1% and H_2_O; 71 ± 0%) which, estimated from the sub-maximal test, equaled approximately 56% of VO_2max_. Athletes were supplied with water only (minimum of 1 mL water·kg^−1^·h^−1^) to deplete muscle glycogen stores during this session. Athletes were randomly assigned to either water (H_2_O group; *n* = 7) or CHO provision (CHO group; 1.06 g CHO·kg^−1^·h^−1^; *n* = 8) formulated for optimal loading for the following 4 h recovery period (Fig.1A). Room temperature (∼22°C) and humidity (∼35%) were maintained stable during the cycling exercise. Subjects cycled using personal shoes, pedals, and bicycles mounted on turbo trainers (Elite Crono Mag ElastoGel Trainer, Fontaniva, Italy) at self-selected cadence.

### CHO supplementation

Dietary intake was controlled and corresponded to the recommendations from the American College of Sports Medicine (Rodriguez et al. 2009). Calorie intake was calculated on basis of body mass. Breakfast consisted of CHO rich foods (i.e. porridge oats, raisins, skimmed milk, orange juice and energy bar; 82 kJ·kg^−1^·bw). Following the prolonged exercise, the CHO group received a meal consisting of pasta, chicken, vegetables and a CHO beverage (1.07 g CHO·kg·bw^−1^·h^−1^) during the initial 2 h of the recovery period and a CHO beverage and an energy bar (1.05 g CHO·kg·bw^−1^·h^−1^) during the subsequent 2 h of the recovery. The H_2_O group was provided with water only during the initial 4 h of the recovery period. The remaining 20 h of recovery both groups received standardized CHO-enriched meals (CHO: dinner and breakfast; H_2_O: lunch, sportsbar, dinner and breakfast) equalizing the total energy intake between groups. The time elapsed between breakfast consumption and collection of pre and 24 h-biopsies was 1.5 h on both days. Subjects received 264 kJ·kg·bw^−1^ on the first experimental day corresponding to 17.2 to 22.6 MJ (≈10 g CHO kg·bw^−1^·day^−1^).

### Sample collection

Muscle biopsies were collected from *m. vastus lateralis* under local anesthetic (1% lidocaine; Amgros, Copenhagen, Denmark) using a 5 mm Bergström needle with suction. Samples were collected before (pre) and approximately 5 min after the exercise bout (post), as well as after 4 h and 24 h of recovery (4, 24 h). For controls only the pre samples were collected. For all subjects the experimental procedure was initiated in the morning according to our standardized protocol. Participants were instructed to avoid physical activity (24 h), alcohol and caffeine (72 h) before the experiment and were provided with a standardized diet during the entire experimental period (24 h pre and post exercise). Biopsies were taken in the same region and depth and care was taken to avoid damaging effect of multiple biopsies by alternating legs and separating incisions by ∼5 cm (Vissing et al. 2005). Of 100–150 mg tissue was excised, quickly dissected from fat and connective tissue, divided into multiple pieces and either directly frozen in liquid nitrogen or embedded in TissueTek (Sakura Finetek, Alphen aan den Rijn, the Netherlands) and frozen in precooled isopentane. The samples were stored at −80°C for further analysis.

### MHC isoform analysis

Whole muscle homogenate and gel electrophoresis was used to determine the MHC isoform composition as previously described in (Salviati et al. 1986) and modified for humans (Andersen and Aagaard 2000). Briefly, muscle biopsy samples were manually homogenized in 1:10 volumes of ice-cold buffer (300 sucrose m·mol·L^−1^, 1 EDTA m·mol·L^−1^, 10 m·mol·L^−1^ NaN3, 40 m·mol·L^−1^ Tris-base and 40 m·mol·L^−1^L-histidine, pH 7.8) at 0°C, and subsequently frozen in liquid nitrogen. For analysis, homogenates were mixed with sample-buffer (10% glycerol, 5% 2-mercaptoethanol and 2.3% SDS, 62.5 m·mol·L^−1^ Tris and 0.2% bromophenolblue, pH 6.8), heated at 100°C for 3 min and for each three samples (9 mg, 16 mg and 22 mg protein) were loaded on a SDS-PAGE gel (6% polyacrylamide). Gels were run at 80 V for at least 60 h at 4°C and bands were stained with Coomassie. The gels were scanned (Linoscan 1400 scanner, Heidelberg, Germany) and MHC bands quantified densitometrically (Phoretix 1D, nonlinear, Newcastle, UK) according to the loaded protein amounts. Western blotting was used to identify MHC II (Sigma M 4276) with the protocol Xcell IITM (Invitrogen, Carlsbad, CA).

### Muscle glycogen content

The data on muscle glycogen content have previously been published (Gejl et al. 2014). Muscle glycogen was determined using spectrophotometry (Beckman DU 650) (Passonneau and Lowry 1993). Freeze dried muscle tissue was boiled in 3% w/v of 1 mol·L^−1^ HCL for 2.5 h before it was cooled and centrifuged at 3500 *g* for 10 min at 4°C. One milliliter of reagent solution (1 mol·L^−1^ tris-buffer, 100 m·mol·L^−1^ ATP, 1 mol·L^−1^ MgCl_2_, 100 m·mol·L^−1^ NADP^+^ and G-6-PDH) was mixed with 40 *μ*L of boiled muscle sample and the process was initiated by adding 10 *μ*L of diluted hexokinase. Absorbance was recorded for 60 min and muscle glycogen expressed as mmol·kg^−1^·dw^−1^. The analysis was unsuccessful in four of the muscle biopsies, which forced us to leave out these data points.

### Periodic acid-Schiff (PAS) staining with immunofluorescences

Sections were stained with PAS and immunofluorescence for myosin heavy chain I with a procedure adopted from Schaart et al. (2004). All incubations were carried out at room temperature (19–22°C). Briefly, muscle sections were fixed for 10 min in 4% formaldehyde with 0.05% Triton X-100, washed and then treated with 1% periodic acid (Sigma Aldrich, Brøndby, Denmark) in water for 5 min. The slides were incubated with Schiff's reagent (Sigma Aldrich; containing 1% pararosaniline, 4% sodium metabisulphate and 0.25 mol·L^−1^ hydrochloric acid) for 15 min and washed in water for 10 min. For immunofluorescence staining, sections were washed in Wash Buffer (Dako, Glostrup, Denmark) and blocked in Protein Block (Dako) before incubation with monoclonal anti-myosin heavy chain I (MHC-I), 1:2000 (Sigma-Aldrich) for 1 h and subsequent Alexa-flour 488 donkey anti-mouse secondary antibody, 1:500 for 1 h. The slides were mounted with ProLong Gold Antifade with dapi (Life Technologies, Naerum, Denmark).

### RNA extraction and cDNA synthesis

Tissue samples were weighed and homogenized in tubes containing 10 ceramic beads and one silicium crystal using the MagnaLyzer (Roche, Hvidovre, Denmark). Subsequent, total RNA was extracted using TRIzol Reagent (Life Technologies) according to the manufactures directions and RNA concentration was measured using the Nano Drop (ND1000; Thermo Scientific, Hvidovre, Denmark) returning 260/280 ratios above 1.8 for all samples. 500 ng of RNA was converted into complementary DNA using High Capacity cDNA Reverse Transcription Kit (Applied Biosystems, Naerum, Denmark).

### Real-time reverse transcriptase Polymerase Chain Reaction

Real-time RT-PCR was performed using TaqMan Low Density Arrays custom designed with 46 genes and two controls. Each port on the cards was loaded with cDNA equivalent to 125 ng total RNA mixed with 2× Gene Expression Mastermix and samples were run at 50 cycles in duplicates on the 7900 Sequence Detection System (all reagents from Applied Biosystems). Data were collected and analyzed using SDS 2.4 software (Applied Biosystems). Technical duplicates were evaluated and samples excluded when Δ*C*_*t*_ > 1. Control genes were verified using GeNorm software (Vandesompele et al. 2002) and data were expressed relative to the reference genes GAPDH and RPLP0 using the qBase+ software (Biogazelle, Zwijnaarde, Belguim). The following TaqMan assays were used; PPARGC1A-Hs01016724_m1, NRF1-Hs00602161_m1, TFAM-Hs01082775_m1, COX4I1-Hs00971639_m1, PDK4-Hs01037712_m1, LPL-Hs00173425_m1, PPARA-Hs00947538_m1, UCP3-Hs01106052_m1, SLC2A4-Hs00168966_m1, RPLP0-Hs99999902_m1, GAPDH-Hs99999905_m1.

### PGC-1α protein expression

Eight *μ*m cryo-sections were fixed in 4% NBF, permeablized in 0.5% triton 100-X, blocked in protein blocker (Dako) and incubated with (1) PGC-1 alone (K-15, 1:200; Santa Cruz) (Russell et al. 2003); (2) PGC-1 together with monoclonal anti-myosin heavy chain I (MHC-I, 1:10.000; Sigma-Aldrich) or (3) PGC-1 together with monoclonal anti-myosin heavy chain II (MHC-II, 1:10.000; Sigma-Aldrich) over night. Subsequent, sections were incubated with Alexa-flour 555 donkey anti-mouse and 488 goat anti-rabbit secondary antibodies (Life Technologies), 1:1000 for 1 h and mounted with ProLong Gold Antifade with dapi (Life Technologies). Negative controls leaving out primary antibodies were included.

### Statistical analysis

Before performing the statistical analysis, all gene expression data were logarithmic transformed to insure normal distribution and are reported in the article as geometric mean ± SEM (back transformed values) of the expression. The remaining data are presented as mean ± SE. A two-way ANOVA (SigmaStat, 3.5; Systat Software, Erkrath, Germany) was performed between *time* and *treatment* (CHO or H_2_O) for mRNA targets and glycogen followed by a Student-Newman-Keuls post hoc test, when a significant interaction was identified. Student's t-test was used to identify differences in baseline mRNA, and Pearsons correlation was used for correlations. Data were considered significant when *P* < 0.05. Graphs were made employing GraphPad Prism 5.0 (GraphPad Software, Inc., La Jolla, CA).

## Results

### Fiber type distribution

The HT athletes displayed an increased number of type I fibers compared to the UT (65.0 ± 3.1% vs. 48.3 ± 5.3%, *P* = 0.010) with a consequent lower proportion of type II fibers (35.0 ± 3.3% vs. 51.7 ± 5.3%, Table1). The fiber type distribution in the CHO and H_2_O groups were not identical as CHO had 73.5.3 ± 2.7% type I fibers compared to 55.4 ± 3.2% in H_2_O (*P* = 0.001, Table1).

### Baseline mRNA expression

**Figure 2:**
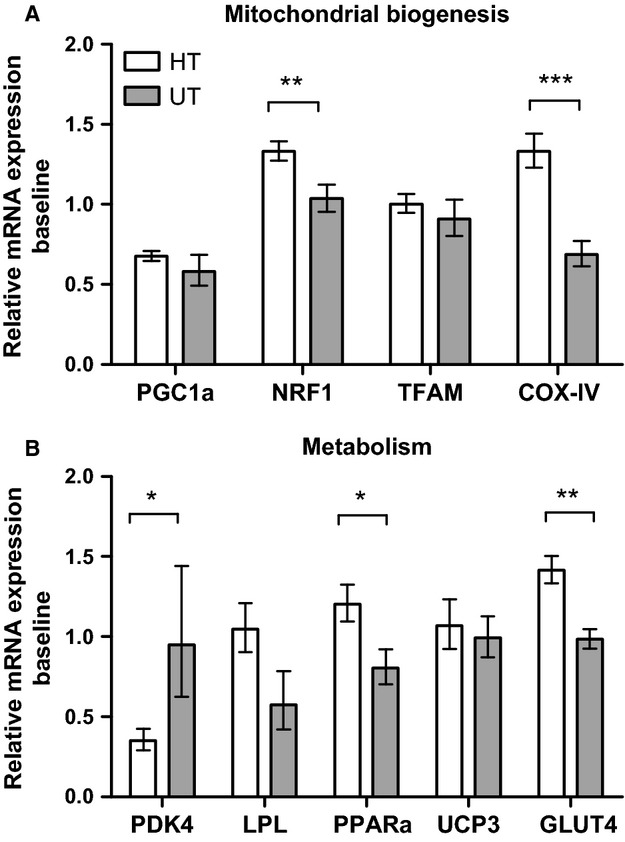
Baseline mRNA expression levels of HT vs. UT. Expressions levels of targets related to mitochondrial biogenesis (A) or metabolism (B). Values are normalized to the reference genes GAPDH and RPLP0, and reported as geometric means ± SEM. HT; *n* = 15, UT; *n* = 8. Significantly different from UT; *(*P* < 0.05), **(*P* < 0.01), ***(*P* < 0.001).

**Figure 3:**
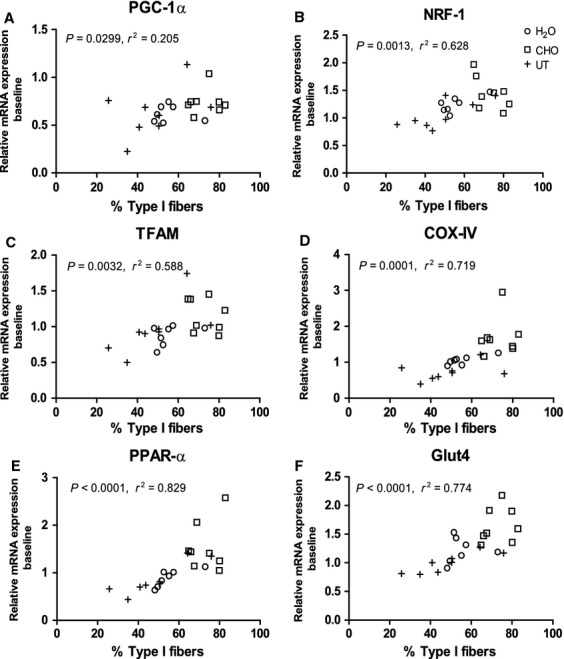
Baseline mRNA expression correlates with percentage of type I fibers. Correlations between individual percent of type I fibers and baseline mRNA expression (A–F). H_2_O: white circle; CHO: white square; UT: black cross. Values are normalized to the reference genes GAPDH and RPLP0.

Comparing baseline mRNA expressions showed a higher level of expression in HT subjects of nuclear respiratory factor 1 (NRF-1) and cytochrome c oxidase-IV (COX-IV) involved in mitochondrial biogenesis (29 and 94%, respectively) (Fig.2A). Baseline mRNA of metabolic targets pyruvate dehydrogenase kinase 4 (PDK4) was lower (63%), while glucose transporter type 4 (GLUT4), and peroxisome proliferator-activated receptor alpha (PPAR*α*) was higher (63 and 44%, respectively) in HT compared to the UT (Fig.2B). When combining data from the HT and the UT percentage of type I fibers and baseline mRNA expression were positively correlated for PGC-1*α*, TFAM, NRF1, COX-IV, PPAR-*α* and GLUT4 (*P* < 0.05, Fig.3A–F).

### Glycogen

**Figure 4:**
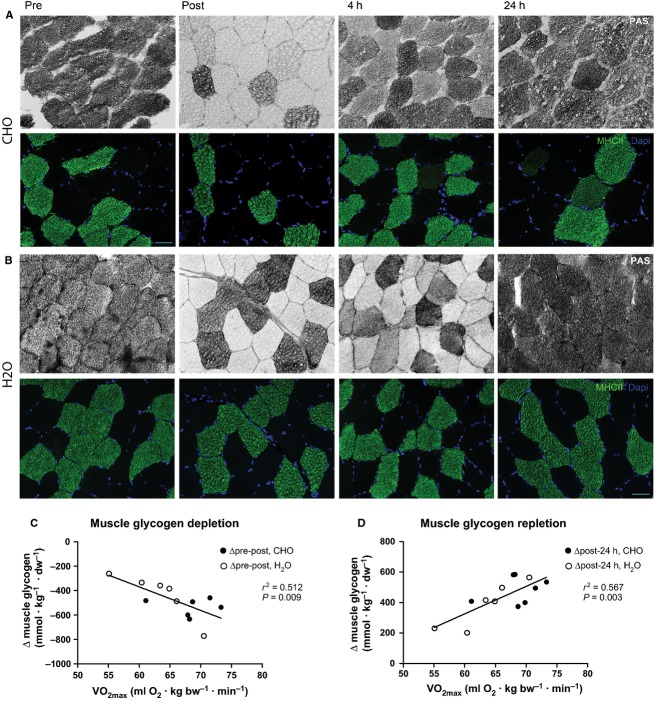
Selective muscle glycogen depletion. Double immunohistochemical stainings show effective glycogen depletion post exercise, primarily of type I fibers and restoration of glycogen after 4 and 24 h of recovery. White fibers indicate glycogen depletion (top), while type II fibers are labeled green and nuclei blue (bottom). Images are from a subject in CHO (A) and H_2_O group (B). Scale bar: 50 *μ*m. Absolut changes in muscle glycogen correlated with VO_2max_ for pre-post exercise depletion (*y* = −19.3*x* + 788.3; *r*^2^ = 0.5123; *P* = 0.0089; *n* = 12) and post-24 h repletion (*y* = 18.0*x* − 755.9; *r*^2^ = 0.5673; *P* = 0.0030; *n* = 13) (C, D).

Biochemical determination of muscle glycogen is given in Table2. Periodic acid-Schiff stainings on transverse muscle biopsy sections revealed a marked reduction in muscle glycogen with depletion primarily of type I fibers (Fig.4A). Additionally, absolute changes in muscle glycogen from both groups during depletion (pre-post) and replenishment (post-24 h) correlated positively with VO_2max_ (*r*^2^ = 0.512, *P* = 0.009 and *r*^2^ = 0.567, *P* = 0.003 for depletion and repletion, respectively; Fig.4B and C).

**Table 2:** Muscle glycogen levels. Total muscle glycogen concentrations at pre and post exercise and after 4 and 24 h of recovery expressed as mmol·kg^−1^·dw^−1^. Note different glycogen repletion rates between H_2_O and CHO after 4 h recovery.

	CHO (mmol·kg^−1^·dw^−1^)	H_2_O (mmol·kg^−1^·dw^−1^)
Pre	732 ± 28	666 ± 43
Post	234 ± 39***	245 ± 49***
4 h	444 ± 24***	264 ± 31***^,^†
24 h	704 ± 39	616 ± 31

Significantly different from pre is indicated by ^*^^*^^*^ (*P* < 0.001), and by ^†^when significantly different from CHO at corresponding time point (*P* = 0.002).

### Gene response to exercise

**Figure 5:**
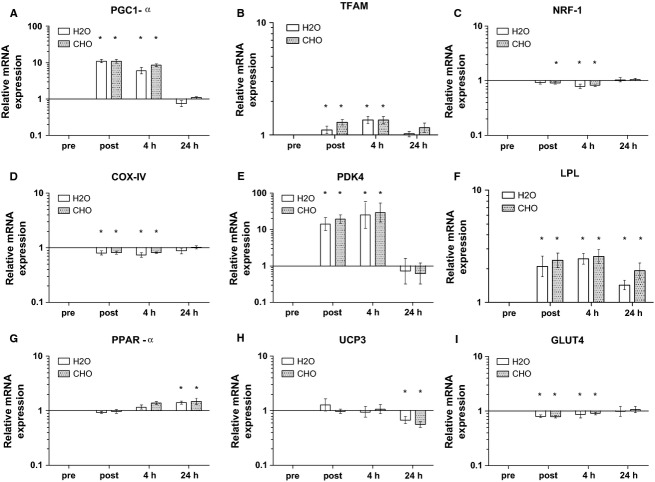
Mitochondrial biogenesis and metabolic mRNA expression in H2O and CHO. Values are normalized to the reference genes GAPDH and RPLP0, expressed relative to pre values, and reported as geometric means ± SEM, *n* = 15. Significantly different from pre is indicated by *(*P* < 0.05).

Exercise induced changes in mRNA expression are depicted in Fig.5. PGC-1*α* mRNA was increased 11 fold immediately after the exercise bout and remained elevated for the first 4 h in recovery, while returning to baseline levels by 24 h. Likewise, TFAM mRNA expression showed a significant increase post exercise and at 4 h, while NRF1 and COX-IV mRNA showed a significant decrease at those time points. All three genes returned to pre levels by 24 h (Fig.5).

A significant increase in mRNA expression was found for PDK4 and lipoprotein lipase (LPL) post and 4 h after exercise, while PDK4 returned to baseline levels LPL was still increased after 24 h (Fig.5). The exercise bout did not give rise to an immediate effect on PPAR-*α* or mitochondrial uncoupling protein 3 (UCP3) mRNA post or at 4 h, although an increase and decrease, respectively was found after 24 h. GLUT4 mRNA was slightly decreased post exercise, but returned to basal levels during the recovery period.

### Influence of CHO vs. H_2_O on gene expression

Supplementation with CHO during 4 h of recovery did not increased expression of any of the mRNA targets after 4 h compared to H_2_O group (Fig.5).

### PGC-1α protein expression

**Figure 6:**
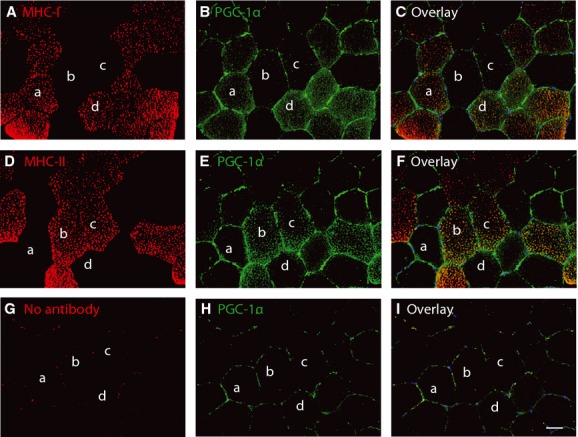
Immunohistochemical evaluation of PGC-1*α* protein expression. Serial sections from the 4 h time point are stained with PGC-1*α* and MHC-I (A–C), PGC-1*α* and MHC-II (D–F) or PGC-1*α* alone (G–I). Scale bar: 50 *μ*m.

Immunohistochemical evaluation of PGC1-*α* protein showed inconsistent results (Fig.6). Evaluation of PGC-1*α* alone revealed no positivity, whereas double labelling with PGC-1*α* and MHC-I showed PGC-1*α*-positive type I fibers, and double labelling of PGC-1*α* and MHC-II showed PGC-1*α*-positive type II fibers. Negative controls showed no staining.

## Discussion

This study is, to our knowledge, the first to investigate the effect of carbohydrate intake versus a delayed replenishment of glycogen stores of more than 2 h following long term endurance exercise on PGC-1*α* and downstream metabolic gene targets in HT athletes. The exercise protocol induced a transient expression in PGC-1*α* and several downstream gene targets; however CHO restriction during recovery did not increase the gene expression response. Muscle glycogen stores and gene expressions levels were completely recovered after 24 h with and without CHO intake. These major findings demonstrate that (I) delayed replenishment of glycogen stores 4 h into recovery does not augment the expression of PGC-1*α* and downstream metabolic gene targets after glycogen depleting exercise and (II) withholding the initiation of refueling for as long as 4 h does not compromise muscle glycogen content in HT athletes after 24 h of recovery from long term endurance exercise. These observations can influence nutritional strategies in training and competition of athletes that for various reasons cannot refuel immediately, and athletes wishing to manipulate their PGC-1*α* response should not do so by combining nutritional and long term exercise strategies.

### Baseline comparison HT vs. UT

Generally, the HT displayed an increased number of type I fibers, which can be the result of a natural selection of athletes with a high proportion of type I fibers, but also reflect a switch towards fibers with a more oxidative profile (Henriksson 1992). Interestingly, we observed that percentage of type I fibers and baseline mRNA expression were positively correlated, which supports previous findings (Schmitt et al. 2003; Stepto et al. 2009). Similarly, PGC-1*α* mRNA was up-regulated in endurance athletes compared to normal active and spinal cord injured (Kramer et al. 2006) suggesting type I fibers to produce and contain more transcripts potentially due to a higher mitochondrial density and selective activation during endurance exercise. The high basal levels of PPAR*α* and GLUT4 and decreased levels of PDK4 in HT athletes compared to UT suggest that HT possess a highly efficient lipid and glucose metabolisms. The finding that the absolute changes in muscle glycogen were positively correlated with VO_2max_ further underlines the training-induced capability of muscle facing metabolic challenges. Plausible explanations for the variations in glycogen storage capacity could be differences in muscle content of e.g. glut-4 protein (Hickner et al. 1997), insulin signaling (Wittwer et al. 2004) or muscle blood flow (Ebeling et al. 1993) and ultimately glucose distribution to the muscles, but the increased proportion of type I fibers in HT might also influence this finding.

### Exercise induced gene response

The experimental protocol was designed to reflect a regular training bout and consistent with previous studies (Pilegaard et al. 2003; Cluberton et al. 2005; Psilander et al. 2013) we found an increase in PGC-1*α* mRNA following exercise, which returned to baseline levels within 24 h of recovery. The protocol led to an 11-fold increase in PGC-1*α* immediately after the exercise bout, which is analogous to the highest increase reported in the literature (Watt et al. 2004; Wang et al. 2009; Harber et al. 2010), suggesting that long term endurance exercise sessions impact the mitochondrial biogenesis substantially in HT. Furthermore, an accompanying increase in TFAM indicated a direct activation of mitochondrial gene expression and initiation of mitochondrial biogenesis. The exercise bout led to ample increases in both PDK4 and LPL mRNA, proposing a switch from glucose to fatty acid metabolism in order to spare glycogen. This shift has previously been shown following glycogen depleting exercise in untrained (Schmitt et al. 2003; Pilegaard and Neufer 2004), and appears to be similar in the highly endurance trained muscle.

### Influencing PGC-1α response

Following exercise, the muscle glycogen levels were very low and withholding CHO intake during the initial recovery phase induced a marked difference in glycogen repletion rates between CHO and H_2_O groups prompting a potential difference in availability of circulating substrates between groups (e.g. FFA/glucose, insulin, hormones). Nevertheless, in contrast to our hypothesis we found no difference in mRNA expression between CHO and H_2_O. This is in line with other studies finding no effect of CHO restriction during recovery from exercise (Cluberton et al. 2005; Mathai et al. 2008; Cochran et al. 2010).

Differentiating between the effects of low glycogen and exercising with low glycogen can be difficult and might explain some of the difference observed in studies regarding PGC-1*α*. In the present study low glycogen alone did not augment PGC-1*α* expression and other studies supports the need for combining exercise with low glycogen (Hansen et al. 2005; Yeo et al. 2008; Psilander et al. 2013; Bartlett et al. 2013) Duration and intensity might also influence the PGC-1*α* response. In the present study, it is likely that the exercise duration alone impacts the skeletal muscle to such an extent that subsequent dietary interventions are incapable of further activating PGC-1*α*. Several studies investigating “endurance” exercise use protocols of 60–75 min of exercise, and since PGC-1*α* increase in an intensity-dependent manner (Egan et al. 2010), it is important that the exercise protocol reflects regular work of a group of endurance athletes. This is highlighted by the ability of short high intensity interval training (HIT) protocols in inducing moderate PGC-1*α* responses (Little et al. 2011). The present study was designed with the accustomed training regime of endurance athletes in mind, as 4 h cycling is parts of the weekly training program. If the duration/intensity-dependent activated PGC-1*α* increase independently of dietary intake, it is questionable if CHO restriction will have any practical effect in the training regime of the highly endurance trained, who exercises up to 35 h per week.

### Timing of CHO intake

For years athletes have been advised to consume CHO as soon as possible post exercise, and delaying the process might impact the performance within the next 4–8 h (Ivy et al. 1988). This study showed a marked difference in muscle glycogen at 4 h recovery with the H_2_O group being physically affected by deprived glycogen levels. However, after 24 h these differences vanished suggesting that CHO restriction did not influence glycogen recovery, as long as sufficient energy was consumed overall. A previous study lead to a similar conclusion after refraining from CHO for 2 h following exercise (Parkin et al. 1997), and muscle glycogen content 24 h after exercise was identical between subjects consuming a few large or numerous small meals (Costill et al. 1981; Burke et al. 1996). At which point between 4 h and 24 h the H_2_O group “catches up” with the CHO group will be of interest in regard to optimize athletic performance, but answers to this question need to be addressed in subsequent studies.

### Methodological considerations

This study was limited in the number of biopsies that could be collected from each subject. In future experiments elucidation of the mRNA expression levels at time points between 4 and 24 h and beyond would be helpful in understanding the effect of low CHO recovery. The fiber type analysis revealed a difference in fiber type distribution between CHO and H_2_O despite randomizing the subjects to either group. Based on our hypothesis we expected an augmented increase in PGC-1*α* expression in H_2_O upon the nutritional intervention and accompanying metabolic stress. The baseline correlation between type 1 fibers and PGC-1*α* indicates a potential for higher PGC-1*α* expression in CHO, but such a difference was not detected following identical exercise stimulus in the two groups. Further,when normalizing mRNA levels for each subject to the corresponding individual fiber type distribution at every time point, it did not change the mRNA-response, suggesting that neither different baseline potential nor fiber type distributions affected the increase in PGC-1*α*.

Immunohostochemical evaluation of PGC-1*α* protein expression was unsuccessful (Fig.6) and highlights the problems arising when trying to examine of PGC-1*α* at the protein level, as no reliable antibodies exist. However, a general consensus exists that PGC-1*α* is mainly regulated at the transcriptional level (Olesen et al. 2010), and mRNA is, at least for now, the only way to evaluate PGC-1*α* until new antibodies are developed.

## Conclusions

In summary, the results from the present study demonstrated that CHO restriction during the first 4 h following glycogen depleting endurance exercise did not influence the metabolic gene expression, including PGC-1*α*. However, within 24 h all subjects were able to recover to pre exercise levels in terms of glycogen storage and most mRNA targets, suggesting that HT athletes possess the ability to recover relatively fast despite a demanding exercise session followed by a longer period of CHO restriction. This has important potential for athletic nutritional planning in regards to optimizing training and competition outcomes.

Finally, it is important for future reports to investigate fiber type distribution among the included subjects, as this study demonstrates that baseline mRNA values are correlated with fiber type distribution. This may be an important step understanding exercise training physiology and metabolic based diseases such as obesity and type 2 diabetes.

## Conflicts of Interest

No conflicts of interest, financial or otherwise, are declared by the authors.

## References

[b1] Andersen JL, Aagaard P (2000). Myosin heavy chain IIX overshoot in human skeletal muscle. Muscle Nerve.

[b2] Bartlett JD, Louhelainen J, Iqbal Z, Cochran AJ, Gibala MJ, Gregson W (2013). Reduced carbohydrate availability enhances exercise-induced p53 signaling in human skeletal muscle: implications for mitochondrial biogenesis. Am. J. Physiol. Regul. Integr. Comp. Physiol.

[b3] Bergstrom J, Hultman E (1967). Synthesis of muscle glycogen in man after glucose and fructose infusion. Acta Med. Scand.

[b4] Bergstrom J, Hermansen L, Hultman E, Saltin B (1967). Diet, muscle glycogen and physical performance. Acta Physiol. Scand.

[b5] Burke LM, Collier GR, Davis PG, Fricker PA, Sanigorski AJ, Hargreaves M (1996). Muscle glycogen storage after prolonged exercise: effect of the frequency of carbohydrate feedings. Am. J. Clin. Nutr.

[b6] Cluberton LJ, McGee SL, Murphy RM, Hargreaves M (2005). Effect of carbohydrate ingestion on exercise-induced alterations in metabolic gene expression. J. Appl. Physiol.

[b7] Cochran AJ, Little JP, Tarnopolsky MA, Gibala MJ (2010). Carbohydrate feeding during recovery alters the skeletal muscle metabolic response to repeated sessions of high-intensity interval exercise in humans. J. Appl. Physiol.

[b8] Coffey VG, Hawley JA (2007). The molecular bases of training adaptation. Sports Med.

[b9] Costill DL, Sherman WM, Fink WJ, Maresh C, Witten M, Miller JM (1981). The role of dietary carbohydrates in muscle glycogen resynthesis after strenuous running. Am. J. Clin. Nutr.

[b10] Ebeling P, Bourey R, Koranyi L, Tuominen JA, Groop LC, Henriksson J (1993). Mechanism of enhanced insulin sensitivity in athletes. Increased blood flow, muscle glucose transport protein (GLUT-4) concentration, and glycogen synthase activity. J. Clin. Invest.

[b11] Egan B, Carson BP, Garcia-Roves PM, Chibalin AV, Sarsfield FM, Barron N (2010). Exercise intensity-dependent regulation of peroxisome proliferator-activated receptor coactivator-1 mRNA abundance is associated with differential activation of upstream signalling kinases in human skeletal muscle. J. Physiol.

[b12] Erlenbusch M, Haub M, Munoz K, MacConnie S, Stillwell B (2005). Effect of high-fat or high-carbohydrate diets on endurance exercise: a meta-analysis. Int J Sport Nutr Exerc. Metab.

[b13] Gejl KD, Hvid LG, Frandsen U, Jensen K, Sahlin K, Ørtenblad N (2014). Muscle glycogen content modifies SR Ca^2+^ release rate in elite endurance athletes. Med. Sci. Sports Exerc.

[b14] Hansen AK, Fischer CP, Plomgaard P, Andersen JL, Saltin B, Pedersen BK (2005). Skeletal muscle adaptation: training twice every second day vs. training once daily. J. Appl. Physiol.

[b15] Harber MP, Konopka AR, Jemiolo B, Trappe SW, Trappe TA, Reidy PT (2010). Muscle protein synthesis and gene expression during recovery from aerobic exercise in the fasted and fed states. Am. J. Physiol. Regul. Integr. Comp. Physiol.

[b16] Henriksson J (1992). Effects of physical training on the metabolism of skeletal muscle. Diabetes Care.

[b17] Hickner RC, Fisher JS, Hansen PA, Racette SB, Mier CM, Turner MJ (1997). Muscle glycogen accumulation after endurance exercise in trained and untrained individuals. J. Appl. Physiol.

[b18] Ivy JL, Katz AL, Cutler CL, Sherman WM, Coyle EF (1988). Muscle glycogen synthesis after exercise: effect of time of carbohydrate ingestion. J. Appl. Physiol.

[b19] Jacobs I, Kaiser P, Tesch P (1981). Muscle strength and fatigue after selective glycogen depletion in human skeletal muscle fibers. Eur. J. Appl. Physiol. Occup. Physiol.

[b20] Joseph AM, Pilegaard H, Litvintsev A, Leick L, Hood DA (2006). Control of gene expression and mitochondrial biogenesis in the muscular adaptation to endurance exercise. Essays Biochem.

[b21] Kramer DK, Ahlsen M, Norrbom J, Jansson E, Hjeltnes N, Gustafsson T (2006). Human skeletal muscle fibre type variations correlate with PPAR alpha, PPAR delta and PGC-1 alpha mRNA. Acta Physiol. (Oxf.).

[b22] Laursen PB, Jenkins DG (2002). The scientific basis for high-intensity interval training: optimising training programmes and maximising performance in highly trained endurance athletes. Sports Med.

[b23] Little JP, Safdar A, Bishop D, Tarnopolsky MA, Gibala MJ (2011). An acute bout of high-intensity interval training increases the nuclear abundance of PGC-1alpha and activates mitochondrial biogenesis in human skeletal muscle. Am. J. Physiol. Regul. Integr. Comp. Physiol.

[b24] Mathai AS, Bonen A, Benton CR, Robinson DL, Graham TE (2008). Rapid exercise-induced changes in PGC-1alpha mRNA and protein in human skeletal muscle. J. Appl. Physiol.

[b25] Ojuka EO (2004). Role of calcium and AMP kinase in the regulation of mitochondrial biogenesis and GLUT4 levels in muscle. Proc. Nutr. Soc.

[b26] Olesen J, Kiilerich K, Pilegaard H (2010). PGC-1alpha-mediated adaptations in skeletal muscle. Pflugers Arch.

[b27] Parkin JA, Carey MF, Martin IK, Stojanovska L, Febbraio MA (1997). Muscle glycogen storage following prolonged exercise: effect of timing of ingestion of high glycemic index food. Med. Sci. Sports Exerc.

[b28] Passonneau JV, Lowry OH (1993). Enzymatic Analysis, A Practical Guide.

[b29] Pilegaard H, Neufer PD (2004). Transcriptional regulation of pyruvate dehydrogenase kinase 4 in skeletal muscle during and after exercise. Proc. Nutr. Soc.

[b30] Pilegaard H, Ordway GA, Saltin B, Neufer PD (2000). Transcriptional regulation of gene expression in human skeletal muscle during recovery from exercise. Am. J. Physiol. Endocrinol. Metab.

[b31] Pilegaard H, Keller C, Steensberg A, Helge JW, Pedersen BK, Saltin B (2002). Influence of pre-exercise muscle glycogen content on exercise-induced transcriptional regulation of metabolic genes. J. Physiol.

[b32] Pilegaard H, Saltin B, Neufer PD (2003). Exercise induces transient transcriptional activation of the PGC-1alpha gene in human skeletal muscle. J. Physiol.

[b33] Pilegaard H, Osada T, Andersen LT, Helge JW, Saltin B, Neufer PD (2005). Substrate availability and transcriptional regulation of metabolic genes in human skeletal muscle during recovery from exercise. Metabolism.

[b34] Psilander N, Frank P, Flockhart M, Sahlin K (2013). Exercise with low glycogen increases PGC-1alpha gene expression in human skeletal muscle. Eur. J. Appl. Physiol.

[b35] Rodriguez NR, Di Marco NM, Langley S (2009). American College of Sports Medicine position stand. Nutrition and athletic performance. Med. Sci. Sports Exerc.

[b36] Russell AP, Feilchenfeldt J, Schreiber S, Praz M, Crettenand A, Gobelet C (2003). Endurance training in humans leads to fiber type-specific increases in levels of peroxisome proliferator-activated receptor-gamma coactivator-1 and peroxisome proliferator-activated receptor-alpha in skeletal muscle. Diabetes.

[b37] Salviati G, Betto R, Danieli-Betto D, Biasia E, Serena M, Mini M (1986). Myosin light chains and muscle pathology. Neurology.

[b38] Scarpulla RC (2002). Nuclear activators and coactivators in mammalian mitochondrial biogenesis. Biochim. Biophys. Acta.

[b39] Scarpulla RC, Vega RB, Kelly DP (2012). Transcriptional integration of mitochondrial biogenesis. Trends Endocrinol. Metab.

[b40] Schaart G, Hesselink RP, Keizer HA, van Kranenburg G, Drost MR, Hesselink MK (2004). A modified PAS stain combined with immunofluorescence for quantitative analyses of glycogen in muscle sections. Histochem. Cell Biol.

[b41] Schmitt B, Fluck M, Decombaz J, Kreis R, Boesch C, Wittwer M (2003). Transcriptional adaptations of lipid metabolism in tibialis anterior muscle of endurance-trained athletes. Physiol. Genomics.

[b42] Stepto NK, Coffey VG, Carey AL, Ponnampalam AP, Canny BJ, Powell D (2009). Global gene expression in skeletal muscle from well-trained strength and endurance athletes. Med. Sci. Sports Exerc.

[b43] Vandesompele J, De Preter K, Pattyn F, Poppe B, Van Roy N, De Paepe A (2002). Accurate normalization of real-time quantitative RT-PCR data by geometric averaging of multiple internal control genes. Genome Biol.

[b44] Vissing K, Andersen JL, Schjerling P (2005). Are exercise-induced genes induced by exercise?. FASEB J.

[b45] Wang L, Psilander N, Tonkonogi M, Ding S, Sahlin K (2009). Similar expression of oxidative genes after interval and continuous exercise. Med. Sci. Sports Exerc.

[b46] Watt MJ, Southgate RJ, Holmes AG, Febbraio MA (2004). Suppression of plasma free fatty acids upregulates peroxisome proliferator-activated receptor (PPAR) alpha and delta and PPAR coactivator 1alpha in human skeletal muscle, but not lipid regulatory genes. J. Mol. Endocrinol.

[b47] Wittwer M, Billeter R, Hoppeler H, Fluck M (2004). Regulatory gene expression in skeletal muscle of highly endurance-trained humans. Acta Physiol. Scand.

[b48] Yeo WK, Paton CD, Garnham AP, Burke LM, Carey AL, Hawley JA (2008). Skeletal muscle adaptation and performance responses to once a day versus twice every second day endurance training regimens. J. Appl. Physiol.

